# Post-Bader procedure: a long-term follow-up case study

**DOI:** 10.5935/0004-2749.2021-0509

**Published:** 2022-07-04

**Authors:** Nir Erdinest, Dror Ben Ephraim, Naomi London, Itay lavy, Nadav Levinger

**Affiliations:** 1 Department of Ophthalmology, Hadassah-Hebrew University Medical Center, Jerusalem, Israel; 2 Rambam Health Care Campus, Haifa, Israel; 3 Private practice, Jerusalem, Israel; 4 Department of Opthalmology, Enaim Refractive Surgery Center, Jerusalem, Israel

**Keywords:** Keratoconus, Astigmatism, Cornea, Corneal topography, Ophthalmologic surgical procedures, Contact lenses, Dilatation, pathologic, Acuidade visual, Quality of life, Ceratocone, Astigmatismo, Córnea, Topografia da córnea, Procedimentos cirúrgicos oftalmológicos, Lentes de contato, Dilatação patológica, Acuidade visua, Qualidade de vida

## Abstract

Keratoconus is a progressive disorder that manifests as a cone-like steepening of the central or paracentral inferior cornea and irregular stromal thinning. There is a gradual decrease in visual acuity due to corneal asymmetry, irregular astigmatism, and increased optical aberrations, consequently impacting the quality of life. Several procedures have been developed in an attempt to slow or reverse the progression. The Bader procedure, which includes a pattern of incisions around the circumference of the cornea and at the base of the protruding cone, is one such surgery. These incisions penetrate 70-90% of the cornea’s depth. Its goal is to flatten the topography and reduce corneal asymmetry and irregular astigmatism. Though prior research found these to be highly promising, we report a patient who was given contact lenses to restore and maintain his vision while his corneal ectasia and thinning progressed over the following decade.

## Abstract (pt)

O ceratocone é uma doença progressiva que se manifesta como uma elevação semelhante a um cone da cór­nea central ou paracentral inferior e é associada a uma re­dução irregular da espessura do estroma. Há uma diminuição gradual da acuidade visual devido à assimetria da córnea, ao astigmatismo irregular e a um aumento das aberrações ópticas, o que prejudica a qualidade de vida. Foram desenvolvidos vários procedimentos para tentar interromper ou mesmo reverter a evolução da doença. Um deles é o chamado procedimento de Bader, que inclui um padrão de incisões em volta da circunferência da córnea e na base do cone protuberante. Essas incisões penetram até 70%-90% da profundidade da córnea e têm o objetivo de achatar a topografia e diminuir a assimetria da córnea e o astigmatismo irregular. Embora essa técnica seja muito promissora, segundo um estudo anterior, aqui se apresenta o caso de um paciente no qual esses objetivos não foram atingidos. Esse paciente recebeu lentes de contato para restaurar e manter sua visão, enquanto sua ectasia corneana e a redução da espessura progrediram ao longo da década seguinte.

## INTRODUCTION

Keratoconus is a progressive disorder characterized by a cone-like steepening of the central or paracentral inferior cornea, associated with irregular stromal thinning, eventual breaks in Bowman’s layer, and iron depositions in the epithelial basal layers^(^1^)^. There is a gradual decrease in visual acuity due to corneal asymmetry, irregular astigmatism, and increased optical aberrations, consequently impacting the quality of life^(^1^)^. While contact lenses are the mainstay choice for optical correction^(^2^)^, several therapies have been developed to try and reverse or halt progression to avoid partial or complete corneal keratoplasty. These include collagen cross-linking, intra-stromal rings, arcuate keratotomy, circular keratotomy, and a less known procedure called the Bader procedure. While the first four treatments have been well established and studied^(^3^-^5^)^, the latter is a relatively novel procedure introduced in 2013, combining the techniques of the previous two keratotomies^(^3^)^.

**Figure 1:**
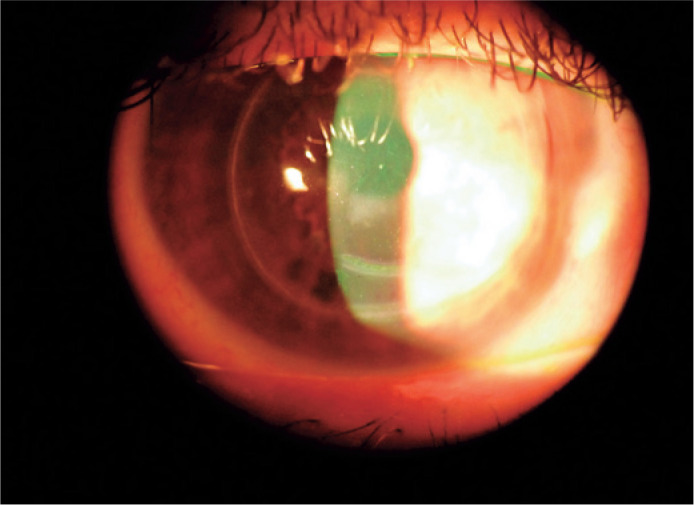
Slit-lamp image of the corneal scars indicating the incisions of the Bader procedure. One incision can be seen around the circumference of the peripheral cornea and two near the base of the ectasia. The white haze beneath the inferior pupil is a long-standing scar.

The Bader procedure is an incisional keratotomy performed under local anesthesia to reduce corneal irregularity^(^3^)^. Depending on the astigmatic power, two equal-length, symmetrical arcuate incisions are made with a Hanna arcitome. They range from 60 degrees for low (1D-3D) astigmatic power to a maximum of 120 degrees for high (more than 7D) astigmatic power^(^4^-^7^)^ and are placed 90 degrees against the steepest meridian, approximately 2.5 mm from the pupillary axis using an arcuate keratome to a depth of 70%-90% depending on the corneal thickness^(^4^-^7^)^ (Figure 1). Next, a 360 degrees circular keratotomy is performed 7 mm-8 mm away from the pupillary axis in two depth levels: 70% depth for the low corneal-power area and 90% depth for the remaining circumference of high corneal power. No sutures are inserted. Immediate post-procedural care consists of topical antibiotics, non-padded shields, collagen and vitamin-C capsules, and topical pilocarpine. The procedure aims to flatten the cornea and induce a natural healing process to reduce asymmetry, irregular astigmatism, and visual dependency on optical aids. The concept that spontaneous healing of the cornea may rectify its topography relies on the finding of a biological source of differentiated keratocytes in the human cornea. However, this was presented in an *in-vitro* study that could not vouch for identical behavior of such cells in the physiological environment to eventually secrete stromal connective tissue^(^8^)^. As a result, the effect’s impact cannot be certain.

A previous study on 14 patients with keratoconus stage III or above presented promising results in a single center by a single surgeon. Primary outcomes were spectacle-corrected visual acuity, refractive error (both dilated and undilated pupils), corneal shape, pachymetry, and indices. Astigmatism decreased in 21 of the 24 treated eyes, corneal volume increased in 22, and visual acuity improved in all eyes by a mean of 59% from before the procedure. The patient shown here underwent this procedure, the outcomes of which were eventually not sustainable, and ultimately he required corneal rigid gas permeable lenses piggybacked on a soft lens to rehabilitate his vision.

## CASE REPORT

The patient is a 37-year-old male diagnosed with keratoconus in his early 20s and remained under this clinic’s care until 2007. His keratoconus was stage IV according to the Krumeich classification at that time, but he was comfortably wearing corneal rigid gas permeable (RGP) lenses for most of his waking hours and had mild dry eye symptoms that were successfully managed with artificial tears as needed. In both eyes, there was a 1.5 mm scar at the corneal apex and minor vertical corneal striae at the base of either side of the cone. At the time of his visit, the parameters of his RGP’s and corrected visual acuity (VA) were keratoconic bi-aspheric design [OD: diameter 8.6 mm, BC 5.90, -10.75D, VA6/9+; OS diameter 9.0 mm, BC 6.25, -10.75D, VA6/9+; OU 6/7+).

Later that year, the patient underwent a Bader procedure in both eyes to potentially eliminate the need for corrective or contact lenses.

According to the operating clinic’s protocol, the patient received collagen (400 mg) and vitamin-C (500 mcg) capsules nightly, and topical G-pilocarpine (2%) was applied every 3 hours during daytime for a total of 6 months. He was then prescribed topical G-pilocarpine (2%) twice a day and was instructed to refrain from contact lens use for two years. He instilled pilocarpine only once a day (in the evenings) and was prescribed glasses two years post-procedure at another clinic (OD 4.75-2.00X175, VA6/50, PH6/18; OS -4.50-2.50X100, VA6/50, PH6/18) and soft toric contact lenses which provided a VA of OD 6/12 and OS 6/15. After a few weeks of dissatisfaction with lenses, he returned to our clinic in 2010 in an attempt to improve his vision.

Fluorescein and lissamine green staining revealed that neither eye had any corneal staining. Conjunctival hyperemia was seen. The initial treatment plan was to fit the patient with a semi-scleral rigid lens to vault the cornea, however, due to the difficulties in acquiring a fitting set, this strategy was discarded. He was fitted with lenses made of FSA (Dk58) material Dia 9.3 mm (OD: BC 5.35 mm, OZ 5.0 mm, Eccentricity 0.9, -17.00D, VA6/7; OS: BC 5.25 mm, OZ 5.0 mm, Eccentricity 1.0, -18.00D, VA 6/7; OU 6/6). The patient wore them comfortably for 8 hours a day without any corneal insult, using artificial tears as needed.

**Figure 2:**
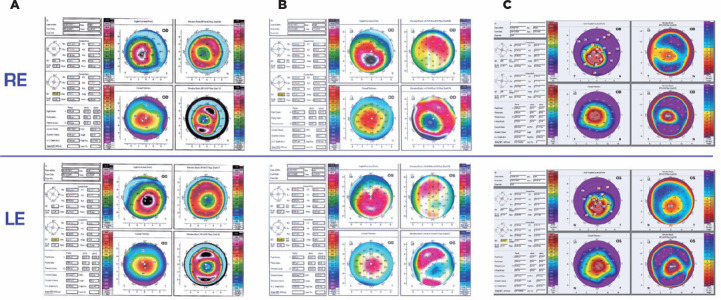
The Pentacam refractive maps in the right and left eyes before the Bader procedure (A), 2 years post-procedure (B), and 13 years post-procedure (C).

The Pentacam (Oculus Wetzlar, Germany) measurements from before the procedure indicated a Q-value that temporarily decreased two years post-procedure. However, afterward, the asphericity increased to above pre-procedure levels. K_max_ continued to elevate, and pachymetry continuously decreased following the procedure during a thirteen year follow-up period (Table 1, Figure 2).

**Table 1:** Pentacam asphericity (Q-value), maximum anterior keratometry radius (Kmax), and pachymetry values before, 2, and 13 years post-Bader procedure

**Pentacam measurement**	**Eye**	**Day before procedure**	**Two years post-procedure**	**Thirteen years post-procedure**
Q-value	Right	-1.30	-0.72	-1.52
	Left	-1.34	-0.27	-1.16
Kmax	Right	62.1D	63.9D	66.7D
	Left	59.4D	60.8D	64.1D
Pachymetry	Right	391 µm	346 µm	310 µm
	Left	410 µm	380 µm	340 µm

**Figure 3:**
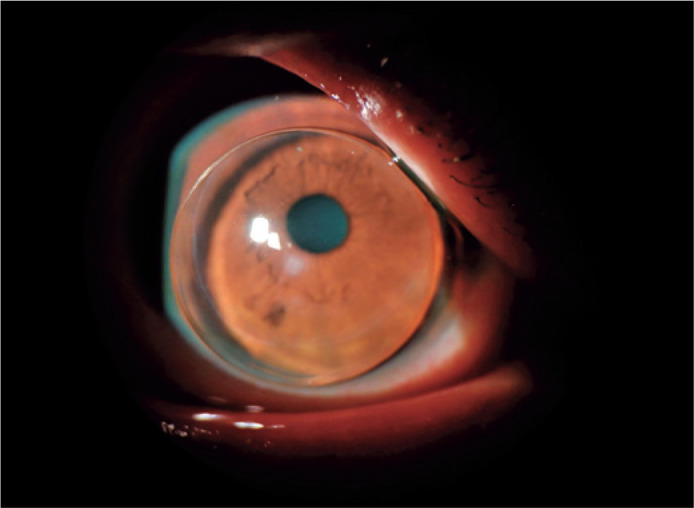
Slit-lamp image of rigid gas permeable lens piggybacked on a daily lens. The incisions from the Bader procedure are visible in the inferior third area of the cornea and the circumference of the peripheral cornea. A long-lasting corneal scar beneath the inferior pupil can be seen as a small white haze through the lens. The rigid lens is piggybacked on a soft daily lens to better center it and protect the protruding apex.

Even though the ectasias had progressed, the apical scars and the corneal striae remained stable. To confirm corneal integrity, fluorescein and lissamine green staining were performed at each follow-up. Cross-linking with this pachymetry is contraindicated. The patient was not interested in scleral lenses and currently utilizes a piggyback modality including a silicone hydrogel daily lens and a Rose K2 (Menicon, Japan) design lens (OD: diameter 8.8 mm, BC 5.1, -21.00D, VA 6/7+; OS: diameter 9.0 mm, BC 5.1, -21.00D, VA 6/7; OU 6/6) (Figure 3).

Corneal integrity has been preserved for over a decade, and progress continues to be carefully monitored, with lenses being modified as needed.

## DISCUSSION

The Bader procedure incorporates a specific pattern of incisions both around the circumference of the cornea and at the base of the ectasia.

According to the advocates of this procedure, the healing process occurs in three stages: a sudden improvement in VA due to the relaxation of the corneal surface; an increase in the fogginess of the patient’s vision due to the production of new “baby cells” produced to fill the troughs created by the procedure, followed by increased corneal thickness secondary to edema that then recedes^(^4^-^7^)^.

We present a case study of a patient who underwent the Bader procedure with a long-term follow-up. Although the operating clinic’s initial follow-up indicated exceptional recovery and even improvement in VA in 100% of the treated eyes^(^3^)^, long-term follow-up at our clinic demonstrated delayed-onset regression and deterioration of his pachymetry over 13 years.

One hypothesis regarding the patient’s deterioration might be related to the partial compliance to pilocarpine treatment beyond six months, which provides a parasympathomimetic effect on corneal epithelial cells, miosis, and reduces the effect of intraocular pressure in the anterior chamber^(^8^-^10^)^. However, healing the epithelium with pilocarpine would take no more than four weeks^(^10^)^, while the patient instilled the drops for several months. Moreover, apart from the patient’s blurred vision and impeded function, long-term use of pilocarpine might cause brow aches, blur from short-term usage, possible permanent miosis, hasten cataract formation, or, while anecdotal, retinal detachments^(^2^,^7^)^.

Another cause of the patient’s deterioration might be the usage of contact lenses, which may interfere with the corneal healing rate. However, this patient struggled with subjective decreased vision for two years, during which he refrained from contact lenses wear. The best-corrected VA now appears to be better than before surgery, although it is unclear whether this is due to the surgery or an improved lens fit.

Sequential Pentacam measurements indicated a progression in corneal thinning and steepening. These changes cannot be conclusively shown to be a direct result of the procedure, nor can such association be completely dismissed.

There is no one explanation for the post-surgical corneal changes that can be assigned or disregarded. It does, however, highlight the necessity for longer, broader follow-up periods and research to determine corneal prognosis.

To the best of our knowledge, this is the first long-term follow-up of a patient following the Bader procedure in the literature. Although the procedure has been reported with encouraging findings after a few years of follow-up, it appears that more extensive studies are needed to confirm the potential long-term effects on the patients’ condition.

Many practitioners may be unfamiliar with the Bader procedure’s unique slit-lamp presentation. While the technique may not accomplish corneal ectasia reduction or independence from optical aid, this case report provides practitioners with means for restoring VA to pre-procedural levels in patients following this novel, less-established procedure, as was done in this case.
